# Neuromonitoring practices for neonates with congenital heart disease: a scoping review

**DOI:** 10.1038/s41390-024-03484-x

**Published:** 2024-08-25

**Authors:** Andrea C. Pardo, Melisa Carrasco, Pia Wintermark, Denise Nunes, Valerie Y. Chock, Shawn Sen, Courtney J. Wusthoff, On behalf of the Newborn Brain Society, Guidelines and Publications Committee, Andrea C. Pardo, Pia Wintermark, Courtney J. Wusthoff, Sonia Bonifacio, Hany Aly, Vann Chau, Hannah Glass, Monica Lemmon, Gabrielle deVeber, James P. Boardman, Dawn Gano, Eric Peeples, Lara M. Leijser, Firdose Nakwa, Thiviya Selvanathan

**Affiliations:** 1 Department of Pediatrics (Neurology and Epilepsy). Northwestern University Feinberg School of Medicine, Chicago, IL US; 2 Department of Neurology. University of Wisconsin School of Medicine and Public Health, Madison, WI US; 3 Department of Pediatrics, Faculty of Medicine and Health Sciences, McGill University, Montreal, Qc Canada; 4 Galter Health Sciences Library. Northwestern University Feinberg School of Medicine, Chicago, IL US; 5 Department of Pediatrics (Neonatology), Lucile Packard Children’s Hospital and Stanford University, Palo Alto, CA US; 6 Department of Pediatrics (Neonatology). Northwestern University Feinberg School of Medicine, Chicago, IL US; 7 Department of Pediatrics, University of California Irvine, Orange, CA US; 8 Department of Neurology, Stanford University, Palo Alto, CA US; 9 Division of Neonatology, Cleveland Clinic Children’s Hospital, Cleveland, OH US; 10 Department of Pediatrics (Neurology), The Hospital for Sick Children, SickKids Research Institute (Neuroscience and Mental Health) and University of Toronto, Toronto, ON Canada; 11 Departments of Neurology & Pediatrics, UCSF School of Medicine, University of California, San Francisco, San Francisco, CA US; 12 Department of Pediatrics, Duke University School of Medicine, Durham, NC USA; 13 Department of Population Health Sciences, Duke University School of Medicine, Durham, NC US; 14 Centre for Reproductive Health, Institute for Regeneration and Repair, University of Edinburgh, Edinburgh, IL UK; 15 Centre for Clinical Brain Sciences, University of Edinburgh, Edinburgh, UK; 16 Department of Pediatrics, Children’s Nebraska, Omaha, NE US; 17 Department of Pediatrics, Section of Neonatology, Cumming School of Medicine, University of Calgary, Calgary, Alberta Canada; 18 Department of Paediatrics and Child Health, Faculty of Health Sciences, University of the Witwatersrand, Johannesburg, South Africa; 19 Department of Pediatrics, BC Children’s Hospital Research Institute and University of British Columbia, Vancouver, BC Canada

## Abstract

**Abstract:**

Neonates with congenital heart disease (CHD) are at risk for adverse neurodevelopmental outcomes. This scoping review summarizes neuromonitoring methods in neonates with CHD. We identified 84 studies investigating the use of near-infrared spectroscopy (NIRS) (*n* = 37), electroencephalography (EEG) (*n* = 20), amplitude-integrated electroencephalography (aEEG) (*n* = 10), transcranial Doppler sonography (TCD) (*n* = 6), and multimodal monitoring (*n* = 11). NIRS was used to evaluate cerebral oxygenation, identify risk thresholds and adverse events in the intensive care unit (ICU), and outcomes. EEG was utilized to screen for seizures and to predict adverse outcomes. Studies of aEEG have focused on characterizing background patterns, detecting seizures, and outcomes. Studies of TCD have focused on correlation with short-term clinical outcomes. Multimodal monitoring studies characterized cerebral physiologic dynamics. Most of the studies were performed in single centers, had a limited number of neonates (range 3–183), demonstrated variability in neuromonitoring practices, and lacked standardized approaches to neurodevelopmental testing. We identified areas of improvement for future research: (1) large multicenter studies to evaluate developmental correlates of neuromonitoring practices; (2) guidelines to standardize neurodevelopmental testing methodologies; (3) research to address geographic variation in resource utilization; (4) integration and synchronization of multimodal monitoring; and (5) research to establish a standardized framework for neuromonitoring techniques across diverse settings.

**Impact:**

- This scoping review summarizes the literature regarding neuromonitoring practices in neonates with congenital heart disease (CHD).
- The identification of low cerebral oxygenation thresholds with NIRS may be used to identify neonates at risk for adverse events in the ICU or adverse neurodevelopmental outcomes.
- Postoperative neuromonitoring with continuous EEG screening for subclinical seizures and status epilepticus, allow for early and appropriate therapy.
- Future studies should focus on enrolling larger multicenter cohorts of neonates with CHD with a standardized framework of neuromonitoring practices in this population.
- Postoperative neurodevelopmental testing should utilize standard assessments and testing intervals.

## Introduction

The global incidence of congenital heart disease (CHD) is reported to be as high as 9.41 per 1000 births and continues to steadily rise^1^. There is a high rate of neurologic complications associated with CHD, which lead to significant morbidities across the lifespan^2–4^. At present, the neurocritical care of neonates with CHD is not standardized. A recent survey identified the use of neuromonitoring techniques, such as near-infrared spectroscopy (NIRS), electroencephalogram (EEG), and amplitude-integrated EEG (aEEG), as areas with significant institutional variability and having the need for further study^5,6^. Specifically, the impact of neuromonitoring practices on the outcomes of neonates with CHD remains understudied. Nevertheless, the American Clinical Neurophysiology Society has identified neonates with CHD as being at high-risk for acute brain injury and has recommended neuromonitoring of this vulnerable population, a recommendation supported by the Neonatal Cardiac Care Collaborative (an academic entity based on a multisociety collaboration aimed to improve outcomes of neonates with congenital heart disease)^7–9^. In support of these and other initiatives focused on improving the neurodevelopmental outcomes of neonates with CHD, we conducted this scoping review in which we sought to synthesize the published literature on neuromonitoring in this population. Specifically, we sought to describe the current state of the field, inform the development of guidelines where appropriate, and investigate gaps in knowledge that may inform future studies of neuromonitoring in neonates with CHD.

## Methods

This study was performed in accordance with the Preferred Reporting Items for Systematic Reviews and Meta-Analyses extension for scoping reviews (PRISMA- ScR) as recommended by the Joanna Briggs Institute (JBI) and PRISMA-ScR reporting guidelines^10^. Search strategies were drafted by an experienced academic medical librarian (DN) and further refined through team discussion (Supplemental Table 1). The following databases were queried: Ovid, Embase, Web of Science and Cochrane. Review objectives, eligibility criteria, and preliminary study characteristics to be extracted were determined a priori and registered into Galter Library Digital Hub, Northwestern Medicine’s institutional open-access scholarly repository^11^. The methodology, inclusion and exclusion criteria, and data extraction proforma were reviewed by the Newborn Brain Society Guidelines and Publications Committee members for feedback. The search results were exported into Covidence systematic review software (Veritas Health Innovation, Melbourne, Australia). All result titles and abstracts were independently reviewed for relevance by authors on the team (AP, MC, PW, SS, VC, CW). Additional full text review of potentially relevant articles was independently performed by two authors. Discrepancies were reviewed by three authors to review for bias and make a final decision about study inclusion (AP, MC, CW). Full text extraction was done by a single author for each article in a pre-specified data proforma (AP, MC, CW, PW) (Supplemental Table 2). Critical appraisal of the articles was done in accordance with JBI guidance on evaluating evidence for each study type and documented in the study proforma in a summative statement^12,13^. We included randomized controlled trials (RCTs), retrospective observational studies, prospective cohorts and studies using other experimental designs published from January 1st,1990 until March 3rd, 2023. We excluded abstracts, conference proceedings, case reports, editorials, letters and other narrative reviews or articles written in languages other than English that were unable to be translated by a member of the study team.

## Results

**Fig. 1:**
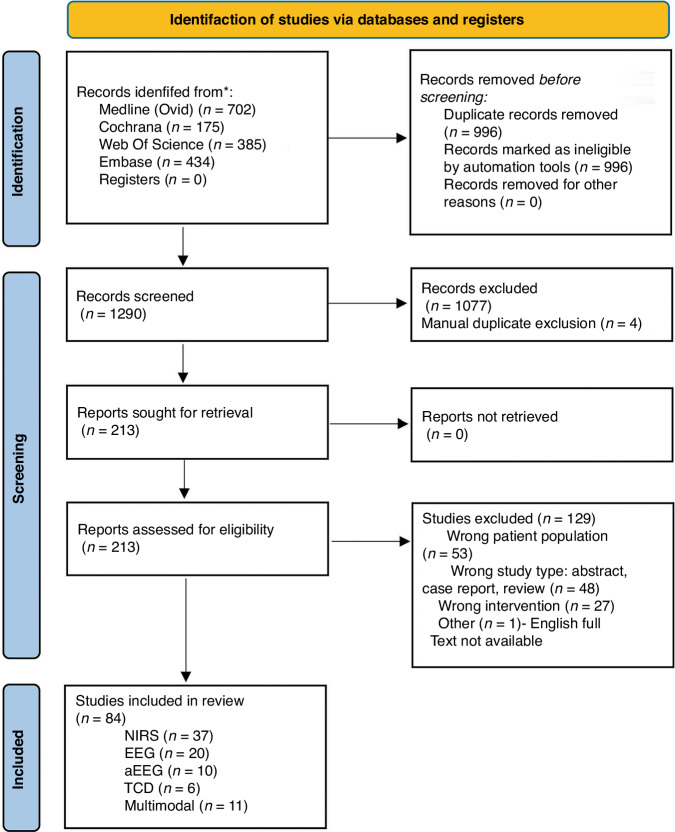
PRISMA diagram. Summary of the screening process.

We screened 1290 published articles, assessed full text of 213 papers for inclusion, and performed full text extraction of 84 articles describing neuromonitoring practices in neonates with CHD. Data were extracted from 37 papers focused on NIRS, 20 on EEG, 10 on aEEG, 6 on transcranial Doppler sonography (TCD) and 11 described the use of two modalities or more (i.e., multimodal monitoring) (Fig. 1). Information describing the neuromonitoring technique, study methodology, number of participants, and type of congenital heart disease for each study is found in Supplemental Table 3. Further study details are available Supplemental File 1.

### Neuromonitoring techniques

#### Near-Infrared Spectroscopy (NIRS)

There were 37 out of 84 studies (44%) that described the use of cerebral NIRS in neonates with CHD. Measurements derived from NIRS included regional cerebral oxygen saturation (rcSO_2_), oxygen extraction fraction (OEF), cerebral blood flow, and cerebral oxygen metabolism (CMRO_2_). Several near infrared spectroscopic instrumentation methodologies were used in the extracted studies. This included both commercially available NIRS as well as frequency domain NIRS (FD-NIRS) and Diffuse Correlation Spectroscopy (DCS), which although only available for research, allows for quantitative measurements of absolute tissue oxygen status measures^14^. We divided the studies evaluating NIRS in neonates with CHD into four groups based on their scope (characterization of NIRS, predictive value for short-term outcomes and adverse events in ICU, predictive value for neurodevelopmental outcome, or correlation with neuroimaging) and these are summarized in Table 1.

**Table 1:** Studies describing the use of NIRS in neonates with CHD.

Scope of study	Number of neonates Mean [range]	Type of congenital heart disease (*n*)			Type of study (*n*)				Findings Summary	Citations
		SVP	TGA	Heterogeneous CHD^a^	Case series	Cohort study	Case-control	Cross-sectional Study		
Validation and characterization of NIRS in CHD (*n* = 21)	56 [3–73]	5	2	14	1	14	6	0	-Measures of rcSO_2_ correlated with low ScvO_2_ but correlation was inconsistent at higher thresholds. -Neonates with CHD had lower rcSO_2_ than healthy neonates. RcSO_2_ did not identify hemodynamically unstable neonates with CHD. -SVP had lower rcSO_2_ than TGA. -Values of rcSO_2_ possibly reflected cerebral autoregulation. -Multiple factors affected rcSO_2._	^15–35^
NIRS as a predictor of short-term outcomes and adverse events in ICU (*n* = 6)	109 [10–194]	3	0	3	0	5	0	1	-Values of rcSO_2_ below a threshold of 50–56% were associated with worse short-term ICU outcomes.	^39–44^
NIRS as predictor of neurodevelopmental outcome (*n* = 6)	45 [28–75]	2	0	4	1	3	1	1	-Postoperative rcSO_2_ below 45–56% were correlated with adverse neurodevelopmental outcomes at different time-points.	^45–50^
NIRS in correlation with neuroimaging (*n* = 4)	44 [32–68]	1	0	3	0	4	0	0	-Measurements of rcSO_2_ did not correlate with new neuroimaging findings after CHD surgery. -NIRS may correlate with ASL.	^51–54^

*NIRS* Near-infrared spectroscopy, *CHD* Congenital Heart Disease, *ASL* arterial spin labeling on MRI, *rcSO2* regional cerebral tissue oxygenation, *SVP* Single ventricle physiology, *TGA* Transposition of the great arteries, *ScvO2* cerebral venous oxyhemoglobin saturation, *ICU* Intensive Care Unit.

^a^Heterogeneous CHD (refers to the inclusion of different phenotypes of CHD in the same study including SVP, TGA, truncus arteriosus, coarctation of the aorta, left ventricular outflow obstruction).

##### Characterization of NIRS in neonates with different phenotypes of CHD (*n* = 21)^15–35^

These studies investigated cohorts ranging from 3 to 73 neonates with CHD monitored prior to surgery or interventional procedure- such as stage 1 palliation or balloon atrial septostomy and continued with postoperative monitoring. NIRS values were obtained as a continuous measurement in most studies, with a majority utilizing commercially available NIRS, and a few utilizing research based NIRS instruments.

Superior vena cava oximetry (ScvO2) serves as a surrogate value of mixed venous oxygen saturation (SvO2) which is an indirect invasive measure of systemic oxygen extraction that has been validated in adults but has conflicting data in children^36,37^. A prior study has demonstrated that lower ScvO2 measures are associated with decreased survival in neonates after palliation of hypoplastic left heart syndrome^38^. Several studies included in this review, aimed to establish the degree of correlation between regional cerebral oxygen saturation (rcSO_2_) and ScvO_2._ Measures of rcSO_2_ accurately correlated with ScvO_2_ at a threshold less than 30%^26^. One study found that, during the preoperative period, rcSO_2_ overestimated ScvO_2_ and underestimated this value in the postoperative period. This study noted that ScvO_2_ was the most accurate in predicting when the venous saturation ranges between 40–60%^27^. Another study reported a poor correlation among rcSO_2_ and ScvO_2_^25^. One study aimed to characterize the rcSO_2_ in left-sided versus right-sided CHD in relation to the change of pulmonary vascular resistance, and found that, among neonates with left-sided CHD, rcSO_2_ measurements were better preserved over time; the authors inferred a decrease in autoregulation in right-sided CHD versus left-sided CHD^15^. One study aimed to describe the correlation of rcSO_2_ and hemodynamic values in the preoperative and postoperative period of single-ventricle palliation with FD-NIRS/DCS measures, reporting that rcSO_2_ did not change when neonates with single-ventricle physiology went from unstable to stable, using a vasoactive inotropic score greater or equal than 10 as a marker of illness severity, despite a change in measured cerebral blood flow and cerebral oxygen metabolism (CMRO_2_), concluding that there is a lack of sensitivity of rcSO_2_ to detect cerebral health^18^. Another study found no change in the comparison of preoperative data with postoperative data utilizing absolute measures obtained with DCS ^16^.

One study noted differences in rcSO_2_ with postural changes in neonates with CHD noting decreased cerebrovascular reactivity in comparison to healthy controls^31^. Although most studies excluded neonates with prematurity, low birth weight, additional cardiac abnormalities and genetic syndromes, usually characterized as high-risk, one study included and compared these neonates defined as high-risk neonates to neonates with CHD and no additional risks, finding no difference in rcSO_2_^20^. Two studies found decreased rcSO_2_ in neonates with CHD when compared to healthy controls^17,19^, and when compared to single-ventricle physiology, biventricular CHD had comparatively higher rcSO_2_^22^. Several factors were noted to alter rcSO_2_ preoperatively: specifically, hemoglobin, hematocrit, and arterial saturation of oxygen^23^. Additionally, NIRS measurements denoted impaired cerebral autoregulation preoperatively in neonates with CHD^35^. In one study, postoperative hemodynamic changes affected rcSO_2_ measurements as well as the arterial content of oxygen and hemoglobin; however, it remains unclear whether optimizing these variables can affect outcomes^23^. Values of rcSO_2_ increased in neonates with transposition of great arteries (TGA) after balloon atrial septostomy ^34^.

Across these studies, rcSO_2_ correlated inconsistently with ScvO_2_, although several systemic factors can affect its measurement, thus making inferences challenging.

##### NIRS as a predictor of short-term outcome and adverse events in the ICU (*n* = 6)^39–44^

In general, rcSO_2_ values below specific thresholds were more likely to be associated with adverse ICU outcomes. This group of studies utilized commercially available NIRS. One study described a threshold of time spent with an rcSO_2_ < 50% and its association with adverse short-term outcome defined by in-hospital mortality, length of stay, delayed sternal closure, need for extracorporeal membrane oxygenation (ECMO), and arrhythmia or bleeding requiring surgical intervention^44^. Similarly, in another study, rcSO_2_ values < 56%, in patients with hypoplastic left heart syndrome (HLHS) after their Norwood procedure, were associated with increased adverse outcomes, defined as in-hospital death, need for ECMO or cardiac intensive care stay greater than 30 days^42^. One study stated that NIRS values predicted adverse events in the cardiac ICU; however, the statistical power of this study was very limited due to the small number of neonates^43^. Another single-center study reviewed the use of NIRS over time and characterized the utilization of NIRS as a tool to effect ventilation changes in neonates with CHD; however, this study characterized changes in practices over time rather than the specific utility of NIRS to influence changes in clinical care^41^. One group developed a multivariate model of rcSO_2_ and hypotension as predictor of in-hospital mortality in neonates with HLHS^40^. Finally, one study utilized NIRS to ascertain readiness for extubation in neonates with CHD after cardiac surgery^39^. Although this was the only multicenter study, NIRS was not systematically used pre- and post-extubation, and the lack of blinding to oximetry measurements may have impacted decision to extubate.

##### NIRS as a predictor of neurodevelopmental outcome (*n* = 6)^45–50^

Most of these studies measured NIRS preoperatively and from 24 h up to 48 h postoperatively. Two of the studies focused specifically on HLHS^46,47^, and the remaining four included a combination of neonates with TGA, truncus arteriosus, coarctation of the aorta and left ventricular flow obstruction, making generalizations across a specific phenotype of CHD difficult. There was wide variability in the neurodevelopmental testing utilized to ascertain neurodevelopmental outcomes. The age of neurodevelopmental testing was variable, and median ages ranged across studies from 6 to 56 months of age. Three studies evaluated outcome with the Bayley Scales of Infant and Toddler Development (BSID). These single-center studies found that reduced cerebral oxygenation was associated with poor neurodevelopmental outcome^45,48,49^. One study noted that, when used in association with lactate values at 24 h postoperatively, a cerebral tissue oxygenation index (cTOI)- an absolute research based NIRS value derived by spectroscopy of oxygenated, reduced and total hemoglobin- below 58% correlated with a Bayley Scales of Infant Development (BSID)-2 score of <70^45^. Another study identified an intraoperative nadir RcSO_2_ threshold of 52% and postoperative nadir threshold of 56% that were correlated with cognitive delay defined as one standard deviation below the normative mean on the BSID-3^48^. One study found that a decreased variability of cTOI was associated with poor neurodevelopmental outcome defined as a mental developmental index or psychomotor developmental index on the BSID-2 < 70^49^. Three other studies evaluated outcomes with different scales such as the Hannover-Wechsler Intelligence Scale, the Cognitive Development for Preschool Age children, the Einstein Neonatal Neurobehavioral Assessment Scale, the Beery-Butkenica VMI test, and the Wechsler Preschool and Primary Scale of Intelligence. This group of studies had more heterogeneous results. One study showed a correlation between preoperative rcSO_2_ and an abnormal (total score more than 7 items outside of the norm) neonatal neurobehavioral assessment during the neonatal period with the Einstein Neonatal Neurobehavioral Assessment Scale; however, this study did not have a long-term follow up^50^. Another study showed that full scale, verbal, and non-verbal intellectual quotient (IQ) at a median of 4.5 years of age were moderately correlated only with the preoperative rcSO_2_, but not the postoperative rcSO_2_ measurements^46^. Lastly, one study showed that the average rcSO_2_ at a threshold lower than 45% was more likely to correlate with an abnormal visual motor integration measured at 56 months^47^. Limitations of these studies included single-center study setting and variable rates of follow up. Importantly, some studies did not account for complications that may have affected outcome outside of the neonatal period, especially considering that neonates with HLHS must undergo multiple stages of correction throughout their childhood.

##### NIRS in correlation with neuroimaging (*n* = 4)^51–54^

All except one study correlating NIRS with neuroimaging included a heterogenous population of neonates with CHD. In general, no rcSO_2_ values or ranges were correlated with new brain injury as assessed by neuroimaging. One study in term neonates with HLHS measured NIRS values both pre-and postoperatively after stage I palliation to investigate whether the values were associated with new or worsening white matter injury (WMI) or periventricular leukomalacia (PVL) on MRI. Although there was a subtle trend for decreasing NIRS measures associated with new WMI/PVL, this did not reach statistical significance. The only factor that determined the presence or absence of WMI/PVL was the time to surgery rather than the rcSO_2_^54^. One study found no association between the total minutes spent with rcSO_2_ below 45% measured by a commercially available NIRS monitor and new MRI injury^51^. This study was the only study that utilized a commercially available NIRS device. Two studies out of the same center, validated the correlation of calculated NIRS measurements, provided by FD-NIRS, with MRI and Arterial Spin Labeling (ASL), an MRI measurement of brain perfusion. They calculated CMRO_2_ values, and noted that despite the small sample size, that there was a linear correlation with rcSO_2_ and the measured MRI indices ^52,53^.

The main limitations of the studies of NIRS and neuroimaging is that they included variable recording times, which likely impacted study associations.

#### Conventional electroencephalography (EEG)

Of the studies included in this review, 20 out of 84 (24%) described the use of conventional continuous EEG in neonates with CHD. The studies were divided into four groups based on their scope and are summarized in Table 2.

**Table 2:** Studies describing the use of EEG in neonates with CHD.

Scope of study	Number of neonates Mean [range]	Type of congenital heart disease (*n*)			Type of study (*n*)			Findings summary	Citations
		SVP	TGA	Heterogeneous CHD^a^	Case series	Cohort study	Other		
EEG background assessment and seizures in neonates with CHD (*n* = 14)	163 [5,1053]	0	4	10	9	1	4	-Association between intraoperative asynchronous burst activity and longer suppression and abnormal postoperative EEG findings. -EEG abnormalities were common postoperatively, mostly characterized as dysmaturity. Sleep features changed postoperatively. -Seizures were common in neonates with CHD, ranging from 4–35% in various cohorts. -Seizures were most commonly subclinical. -Causes of seizures in neonates with CHD included HIE, stroke, or hemorrhages.	^55–68^
EEG as a predictor of adverse events in the intensive care unit (*n* = 2)	20 [18,22]	0	0	2	1	1	0	-EEG could represent a tool to identify changes prior to cardiac arrest in neonates with CHD. -Neonates with CHD and seizures requiring ECMO may require multiple antiseizure medications for seizure control.	^70,71^
EEG as a predictor of long-term outcomes(n = 3)	30 [21,38]	0	1	2	0	3	0	-DHCA and excess discontinuity correlated with lower developmental scores. -Focal seizures postoperatively correlated with the development of focal motor epilepsy.	^72–74^
EEG and neuroimaging correlation (*n* = 1)	16	0	0	1	1	0	0	-Delayed background maturation was associated with injury on MRI.	^75^

*EEG* Electroencephalogram, *SVP* Single ventricle physiology, *TGA* Transposition of the great arteries, *CHD* Congenital Heart Disease, *ECMO* Extracorporeal Membrane Oxygenation, *HIE* Hypoxic Ischemic Encephalopathy, *MRI* Magnetic resonance imaging, *DHCA* Deep hypothermic circulatory arrest,

^a^Heterogeneous CHD (refers to the inclusion of different phenotypes of CHD in the same study including SVP, TGA, truncus arteriosus, coarctation of the aorta, left ventricular outflow obstruction).

##### EEG background assessment and seizures in neonates with CHD (*n* = 14)^55–68^

Several studies sought to characterize the EEG background and presence of seizures in neonates with CHD. Most studies documented the continuous EEG duration. Most studies monitored patients for 24–48 h postoperatively with a few exceptions. One single-center observational cohort study of 40 neonates and monitored neonates 30 min prior to surgery and postoperatively for an unspecified amount of time. This study classified EEG as continuous, normal discontinuous, asynchronous, burst suppression, low voltage burst suppression or with seizures. This study found that neonates with a greater number of intraoperative asynchronous cortical bursts had abnormal postoperative EEG^56^. One study described that two out of 32 neonates developed intraoperative burst pattern with rhythmic sharp, high amplitude asynchronous components and subsequently developed postoperative seizures^55^. Three studies utilized a single TGA dataset (i.e The Boston Circulatory Arrest Study)^61,62,67^. In this cohort of neonates with TGA, the authors found that preoperative EEG abnormalities were common, mostly described as dysmaturity and abnormal background^61^. The monitoring for this cohort started 2 h prior to the surgical time and continued 48 h into the postoperative period and noted an EEG seizure incidence of 20% versus a clinical seizure incidence of 11%. The authors found an association between the length of deep hypothermic circulatory arrest (DHCA) and seizure risk. The authors noted that the longer duration of arrest was associated with a higher risk of EEG seizures and a longer recovery time for return of EEG activity^67^. In this same cohort, seizures were noted to have onset between 13 and 36 h postoperatively, lasting up to 980 min. The risk factors associated with seizures included DHCA, longer duration of circulatory arrest, and diagnosis of ventricular septal defect^62^. One study evaluating EEG tracings for up to 96 h after cardiopulmonary bypass (CPB) noted that 3 of 5 postoperative EEGs were abnormal with tracings that varied between dysmature and very abnormal background. The authors reported that delta power^69^, an electrographic quantitative marker of sleep drive, was persistently below preoperative values in four out of five neonates^57^. An observational cohort study of 77 neonates with heterogenous CHD evaluated pre and postoperative EEG systematically in all neonates undergoing CPB and reported that 69% were normal preoperatively. In the immediate postoperative period, 54% were excessively discontinuous and 33% were noted to have asynchrony and 15% were normal. In this cohort, only 4% of neonates had postoperative seizures^58^. In a larger cohort of 161 neonates with CHD systematically monitored with EEG for 48 h after CPB, none of the clinical events characterized as seizures had an ictal correlate on EEG. However, EEG seizures occurred in 8% of the cohort with a median seizure onset of 20 h after return to the ICU. The seizures were subclinical in most neonates and subclinical status epilepticus was frequent. The most common risk factors associated with seizures were DHCA duration and delayed sternal closure; there was no long-term follow up in this cohort to determine the effect of seizures on neurodevelopment^65^. A prospective observational cohort study of 354 neonates with seizures of all causes further characterized a subgroup of 73 neonates with cardiopulmonary disease with EEG monitoring (including 55 neonates with CHD, one neonate with congenital diaphragmatic hernia, and seven that underwent ECMO). In this group of 55 neonates with no comorbidities other than CHD, 35% had electrographic-only seizures and 22% had status epilepticus. The age at first seizure was 174 h of age (EEG start at 141 h of age), much later than neonates without CHD, whose age at first seizure 21 h of age (EEG onset at 29 h). The etiology of seizures in neonates with CHD was primarily due to hypoxic-ischemic encephalopathy (HIE), ischemic stroke, and intracranial hemorrhage; other less frequent causes included inborn errors of metabolism, neonatal onset epilepsies, and brain malformations^64^. One study investigated a cohort of 70 neonates in a single center that underwent CPB and were monitored with a continuous EEG for 48 h in the postoperative period, reporting seizures in 14% of the neonates^63^. A large cohort of 183 neonates undergoing surgery with CPB for CHD and monitored for 48 h, reported electrographic seizures in 12% of neonates, presenting at a median time of 21 h postoperatively. None had clinical seizures. Phenobarbital administration was associated with a 50% reduction in seizure counts by counting the mean number of seizures per hour in the 3 h period before treatment and comparing with the number of seizures per hour in the 1 h period after treatment. The laterality of seizure onset was associated with major findings on MRI^60^. A single-center study of 87 neonates with CHD who underwent CPB followed by EEG monitoring, although of unknown duration, reported an 18% incidence of acute neurologic event defined as either an EEG seizure or an abnormal neurologic examination or movement disorder, with 7% of neonates having electrographic seizures. Variables associated with acute neurologic events in this cohort included an absolute nucleated red blood cell count greater than 2000 cells, an abnormal preoperative brain study, or a 5-min Apgar score less than 7^59^. One study investigated a single-center series of 5 neonates with TGA who underwent EEG in two periods of 6 h before and after surgery. State dependent variables, such as heart rate (HR), HR variability, respiratory frequency, and the spectral properties of the EEG, were examined between quiet and active sleep. In the postoperative period, the percentage of quiet sleep increased and active sleep decreased compared to preoperatively^68^. A single prospective multicenter study enrolled more than 1,000 neonates with CHD who had EEG for 48 h after CPB. This study described the development and validation of a seizure prediction model across three centers, noting that electrographic seizures occurred in 7% of neonates. However, the model was not able to predict seizures well in the validation cohort ^66^.

These studies were limited in their generalizability in that they were performed in single centers, each with heterogeneous cohorts of neonates with CHD, but generally reported an association between intraoperative asynchronous burst activity and longer suppression and abnormal postoperative EEG findings.

##### EEG as a predictor of adverse events in the intensive care unit (*n* = 2)^70,71^

A retrospective cohort in a single center that sought to systematically monitor neonates in the postoperative period for 48 h, included 22 neonates who suffered cardiac arrest; the authors evaluated characteristics of EEG that preceded cardiac arrest. The EEG was described on a 7-category scale at several time-points prior to cardiac arrest. In this cohort, 41% showed worsening background in the 5 min prior to cardiac arrest as compared to one hour before arrest and the first irreversible EEG change preceded bradycardia by a mean of 2 min 33 s. However, there was no control group to evaluate how the abnormal EEG patterns compared in neonates with CHD without cardiac arrest^70^. An observational cohort study that monitored neonates for 24 h, included 18 neonates with CHD who required ECMO versus no ECMO after repair found that seizure burden was higher in the ECMO group. Most of the neonates in the no ECMO group with seizures responded well to the use of levetiracetam for seizure control, but neonates who required ECMO were more likely to require multiple antiseizure medications after levetiracetam for seizure control ^71^.

In these studies, EEG demonstrated changes prior to cardiac arrest in neonates with CHD. Neonates with CHD and seizures necessitating ECMO may require multiple anti-seizure medications for seizure control.

##### EEG as a predictor of long-term outcomes (*n* = 3)^72–74^

These studies were heterogeneous. In these studies, isoelectric background during DHCA and excess intraoperative discontinuity correlated to lower developmental scores, measured by different approaches. One study noted that 11 out of 21 neonates developed severe burst suppression that progressed to isoelectric during DHCA. At follow up, these neonates had lower Vineland Adaptive Behavior Scale scores at 6 years of age for communication versus neonates without isoelectric EEG during DHCA. In addition, longer duration of DHCA correlated with lower Vineland scores^72^. An observational cohort of neonates with d-TGA from 1978 through 1988 studied neonates who underwent different operative approaches, including 32 with continuous EEG. EEG demonstrated focal seizures in 3/15 neonates with a two-stage d-TGA repair approach, and 1/11 had minor EEG changes without clinical signs following the late switch operation, however, the duration of the EEG was not well described. Due to the small number of patients, outcome correlations were not described but it was noted that some patients in this cohort developed focal motor epilepsy^73^. A single-center cohort study of term neonates with CHD monitored with EEG before open heart surgery, and postoperatively for up to 94 h in patient with preoperative seizures and correlated EEG findings with neurodevelopmental outcomes at 18 months. The authors reported that 16% of neonates (5/31) had preoperative seizures, none of which had a clinical correlate. Although this study monitored most neonates regardless of clinical concerns, neuromonitoring was indicated due to a clinical concern for seizure, encephalopathy, neuromuscular paralysis or an abnormal finding. Seizures were more likely in these neonates (67%) than those in whom neuromonitoring was done without a clinical concern (4%). Excess EEG discontinuity (defined as interburst interval of more than 10 seconds) and preoperative seizures were associated with 18-month adverse outcome assessed with the BSID-3 ^74^.

##### EEG and neuroimaging correlation (*n* = 1)^75^

Finally, a single-center study aiming to correlate neural network connectivity and a 24 h EEG in neonates with CHD prior to surgery, examined neonates with MRI injury and delayed structural and microstructural brain development as defined by Diffusion Tractography Imaging (DTI). In this cohort, neonates with injury had a significantly stronger high frequency beta and gamma frequency band. Neonates with delayed microstructural brain development demonstrated significantly weaker low frequency (delta, theta, alpha frequency band connectivity). They also presented with delayed maturation of EEG background activity defined by greater background discontinuity. Upon visual analysis, neonates without injury had more continuity in the awake and active sleep periods, and decreased time of *Tracé alternans* and *Tracé discontinu*, than neonates with injury ^75^.

#### Amplitude-Integrated Electroencephalography (aEEG)

Out of the studies included, 10/84 (12%) described the use of aEEG. The articles are summarized in Table 3.

**Table 3:** Studies describing use of amplitude-integrated EEG.

Scope of Study	Number of neonates Mean [range]	Type of congenital heart disease (n)			Type of study (*n*)			Findings Summary	Citations
		SVP	TGA	Heterogeneous CHD^a^	Case series	Cohort study	Other		
Characterization of aEEG background in neonates with CHD (*n* = 3)	53 [26,72]	0	0	3	0	3	0	-aEEG background in the postoperative period abnormal in almost half of neonates with CHD. -Seizures frequent and mostly subclinical. -Sleep-wake cycling impacted postoperatively and can be affected by sedatives.	^76–78^
aEEG as a predictor of neurodevelopmental outcome (*n* = 4)	70 [32–150]	0	0	4	0	4	0	-There was a large heterogeneity of tests used to evaluate neurodevelopment and timing of evaluation. -Delayed return of sleep-wake cycling associated with worse neurodevelopmental outcomes. -Conflicting data on whether aEEG seizures were more likely to be associated with impaired development.	^80–83^
aEEG as a predictor injury on neuroimaging and outcome (*n* = 3)	37 [10–76]	0	0	4	0	3	0	-Most studies were underpowered to demonstrate an association between aEEG abnormalities and neuroimaging abnormalities.	^84–86^

*aEEG* Amplitude-integrated electroencephalogram, *SVP* Single ventricle physiology, *TGA* Transposition of the great arteries, *CHD* Congenital Heart Disease.

^a^Heterogeneous CHD (refers to the inclusion of different phenotypes of CHD in the same study including SVP, TGA, truncus arteriosus, coarctation of the aorta, left ventricular outflow obstruction).

##### Characterization of aEEG background in neonates with CHD (*n* = 3)^76–78^

These studies demonstrate an abnormal aEEG background in neonates with CHD in the preoperative and postoperative period. One study evaluated the background from aEEG tracings of neonates with CHD for at least 48 h and up to 72 h of recording after surgery. Neonates were systematically monitored, and tracings were interpreted by the attending neonatologist. The aEEG was classified according to a previously published system by Toet^79^. Severely abnormal patterns were noted in 9/62 neonates, with no difference between cyanotic (TGA) versus acyanotic CHD groups (single ventricle physiology, SVP). Seizures were identified in 12/62 (19%) neonates, including one with status epilepticus. The investigators noted that sleep wake cycling (SWC) was present in 36 neonates (58%) by 72 h of age. Among the monitored neonates, 26 (40%) had a continuous normal voltage background for the entire duration of the recording, and 45% had discontinuous normal voltage at some point during the recording. These neonates did not undergo neurodevelopmental outcome assessment^78^. A retrospective cohort of neonates prenatally diagnosed with CHD underwent aEEG recordings for a median of 72 h starting the recording within the first 3 days of life. This study demonstrated a rate of 15% of subclinical seizures (11/72) presenting on the second day of life. Most neonates had normal background (56%), and 36% had discontinuous normal voltage. Most neonates reached sleep wake cycling by 72 h of recording. An abnormal background was associated with the use of sedatives in a multivariate model^77^. A small cohort study (*n* = 26) evaluated the effect of sedation and analgesia on aEEG on neonates with CHD. The investigators monitored neonates 12 h preoperatively and up to 48 h postoperatively and evaluated outcomes with the BSID-2. The investigators reported no seizures and the return to SWC was within a median of 9 h post-surgery. Patients requiring midazolam had significantly later onset of a normal SWC than those not requiring midazolam. These changes in aEEG background did not correlate with neurodevelopmental outcomes evaluated with BSID-2 ^76^.

##### aEEG as a predictor of neurodevelopmental outcome (*n* = 4)^80–83^

Delayed return of sleep wake cycling was associated to worse neurodevelopmental outcomes. One prospective cohort study of neonates with CHD, undergoing cardiopulmonary bypass for repair, evaluated aEEG tracings using the Toet classification system, recording at least 12 h prior to surgery and up to 48 h postoperatively. The study sought to ascertain the association of aEEG with neurodevelopmental outcome at 1 year of age as measured by the Bayley Scale of Infant Development-2 (BSID-2) and at 4 years of age with several different neuropsychologic tests (Wechsler Preschool & Primary Scale of Intelligence, Snijders Omen and Kaufman). The investigators reported a seizure incidence of 7% on aEEG. Abnormal postoperative background and delayed return of sleep wake cycling were independently associated with a lower IQ by 4 years of age^82^. Another study evaluated a retrospective cohort of 32 neonates with CHD undergoing surgery out of 58 patients that were monitored with aEEG postoperatively for longer than 4 h for any type of major surgery. The neurodevelopmental outcome of these children was evaluated at 3 years of age with the BSID-3. The investigators correlated the background pattern with outcome. There are conflicting data on whether aEEG seizures were more likely to be associated to impaired development. This study noted that those patients that had language delay were more likely to have had cardiac surgery than other types of surgery. The study was underpowered to investigate other risk factors and no other differences were found between groups^83^. One study evaluated the aEEG background in neonates undergoing a Norwood type palliation for SVP. Tracing recording began one hour preoperatively and continued for up to 72 h postoperatively. Neurodevelopmental outcome was assessed at 2 years with the BSID-3. The investigators found that 13/39 (33%) neonates had perioperative seizures (9 intraoperatively, 7 postoperative) and these were associated with higher and earlier mortality and impaired development. Recovery to a continuous aEEG background after CPB occurred within 24 h in 62% of this cohort and by 48 h in 74%^81^. A study describing a heterogeneous and larger neonatal CHD population (*n* = 150), noted that 30% of neonates had perioperative seizures on aEEG; however, of the latter, only 25% of them had a clinical correlate. The investigators noted that aEEG seizures were not associated with 2-year outcome. They noted that failure of the aEEG to recover to a continuous background by 48 h was associated with impairment in all outcome domains of the BSID-3. In addition, in this study, postoperative ECMO was associated with delayed aEEG recovery versus those who did not require ECMO^80^. In these studies, there was large heterogeneity of tests utilized to evaluate neurodevelopment and timing of evaluation.

##### aEEG as a predictor of injury on neuroimaging and outcome (*n* = 3)^84–86^

A small cohort study investigated the use of aEEG in 10 acutely ill neonates with CHD, reporting that aEEG was predominantly normal in 5, moderately abnormal in 4 and severely abnormal in 1 neonate. One infant had electrographic seizures, although there was clinical concern for seizures in 6 neonates in this cohort. The study was underpowered to draw conclusions regarding correlation of aEEG and MR abnormalities or neurodevelopmental outcome^85^. In a study of two historical cohorts of neonates with CHD that were monitored starting 6 h prior to surgery and ending 48 h after, neonates with abnormal aEEG background patterns 12–48 h after surgery or with ictal discharges were more likely to demonstrate new brain injury in the postoperative period as characterized by either 1.5 T or 3 T MRI scans. The investigators reported that in this cohort of 76 neonates, 17% had ictal discharges, and 24% had an abnormal background pattern. This study also reviewed recovery to continuous normal voltage background and sleep-wake cycling, but no correlation between aEEG recovery and outcome was found^84^. One study sought to ascertain the association of preoperative aEEG and preoperative MRI findings in CHD. 15/24 neonates had an abnormal aEEG on the first day of recording. Half of the neonates had an abnormal preoperative MRI; however, the study was underpowered to demonstrate a statistically significant association^86^. These studies were small and likely underpowered to find an association between aEEG and MRI findings in neonates with CHD.

#### Transcranial doppler ultrasound (TCD)

Out of the extracted manuscripts, 6/84 (7%) described the use of transcranial Doppler ultrasound (TCD) in neonates with CHD. The articles are summarized in Table 4.

**Table 4:** Articles describing the use of transcranial Doppler ultrasound in neonates with CHD

Number of neonates Mean [range]	Type of congenital heart disease (*n*)			Type of study (*n*)			Findings Summary	Citations
	SVP	TGA	Heterogeneous CHD^a^	Case control Study	Cohort study	Other		
30 [16,47]	2	1	3	2	2	2	-TCD studies were heterogeneous and were not consistently associated with neurodevelopmental outcomes	^87–92^

*SVP* Single ventricle physiology, *TGA* Transposition of the great arteries, *CHD* Congenital Heart Disease, *TCD* transcranial Doppler ultrasound

^a^Heterogeneous CHD (refers to the inclusion of different phenotypes of CHD in the same study including SVP, TGA, truncus arteriosus, coarctation of the aorta, left ventricular outflow obstruction.

There is a limited number of studies evaluating the role of TCD in neonates with CHD. A study in 9 neonates with HLHS undergoing either bilateral pulmonary artery banding or undergoing Norwood operation prior to Glenn repair found that bilateral pulmonary artery banding did not increase cerebral blood flow velocities measured in the proximal middle cerebral arteries (MCA) in the first week after the procedure, and oxygenation balance index (an index to indicate demand-supply balance between the lower and upper body) was lower than in the neonates with the Norwood operation, indicating impaired cerebral perfusion^87^. A cohort study in 28 neonates with TGA undergoing arterial switch procedure evaluated the limits of detectable blood flow in the MCA during low-flow CPB; all had detectable MCA perfusion at flow ≥30 ml/kg/min^88^. A study of 16 neonates with heterogenous CHD described the resistive indices (RI) of major cerebral arteries (anterior and middle cerebral arteries bilaterally) and highlighted that higher RI measured immediately postoperatively correlated with improved cognitive, language and motor outcome on the BSID-3 and the Vineland Adaptive Behavior Scales at 12 months following surgery; however, RI values prior to discharge did not correlate with outcomes^89^. A cohort of 28 neonates with heterogeneous CHD demonstrated that there was no correlation between the postoperative RI and the ability to start enteral feedings or wean mechanical ventilation^90^. A study compared MCA velocities in neonates with TGA to non-neonatal groups with other CHDs who were all randomized to a trial of hemodilution during surgery and showed that absolute values of cerebral blood flow velocity were lower and RI higher in the TGA group at 6 and 18 h after surgery; these indices did not correlate with neurodevelopmental outcome at 1 year of age. Of note, data capture was performed during the induction of anesthesia and did not account for the effect of inhalation anesthetics on cerebral blood flow velocity values^91^. A single-center case-control study in 18 neonates with HLHS versus 6 healthy age-matched controls found that the hybrid stage 1 procedure did not improve the abnormally reduced middle cerebral artery blood flow velocities measured by TCD in neonates with HLHS, and there was no correlation between MCA blood flow velocities and neurodevelopmental outcome assessed at 6 months with BSID-3^92^. Overall, TCD studies were heterogeneous and were not consistently associated with neurodevelopmental outcomes.

#### Multimodal monitoring (use of ≥2 neuromonitoring techniques)

In the extracted manuscripts, 13% (11/84) reviewed the use of more than 2 neuromonitoring techniques in neonates with CHD. The studies are summarized in Table 5.

**Table 5:** Articles describing use of multimodal monitoring in neonates with CHD.

Number of neonates Mean [range]	Type of congenital heart disease (n)			Type of study (n)			Summary of findings	Citations
	SVP	TGA	Heterogeneous CHD^a^	Case series	Cohort study	Other		
41 [10,110]	2	2	7	2	3	6	-There was no consistent association between multimodal monitoring measures and neurodevelopmental outcome. -A severely abnormal aEEG was predictive of a markedly abnormal EEG.	^93–102,106^

*EEG* electroencephalogram, *CHD* Congenital Heart Disease, *aEEG* Amplitude-integrated electroencephalogram, *SVP* Single ventricle physiology, *TGA* Transposition of the great arteries.

^a^Heterogeneous CHD (refers to the inclusion of different phenotypes of CHD in the same study including SVP, TGA, truncus arteriosus, coarctation of the aorta, left ventricular outflow obstruction.

In the studies using ≥2 neuromonitoring modalities, the combined modalities were variable and often not timed-locked. A single-center case-control study in 5 neonates with HLHS undergoing the Norwood-Sano stage I palliation correlated NIRS measurements with cerebral blood volume (CBV) measured by ultrafast power Doppler imaging and showed that CBV was slightly increased postoperatively and recovered 1–2 weeks after surgery, compared to age-matched healthy controls; there was no new MR brain injuries in all except one neonate^93^. One study compared frequency domain (FD-NIRS) and diffuse correlation spectroscopy (DCS) findings in neonates with biventricular circulation, TGA and SVP and healthy controls and demonstrated that neonates with TGA had a postoperative increase in oxygen saturation with a decrease in fractional tissue oxygen extraction (FTOE) and decreased CMRO_2_; only neonates with SVP demonstrated decreased cerebral blood flow index, likely due to secondary diminished cardiac output, oxygen supply and decreased metabolic demand^94^. A cohort study of 27 neonates with CHD undergoing the Norwood procedure either with a Blalock-Taussig shunt or an RV-PA conduit evaluated the correlation between NIRS and TCDs and found that the cerebral diastolic blood flow velocity did not differ significantly between groups intra- and postoperatively, but the cerebral systolic blood flow velocity was higher over time in the Blalock-Taussig shunt group. There were no significant differences in rcSO_2_ between groups at baseline or during bypass^95^. Another study of neonates with TGA undergoing arterial switch operation by CPB with and without DHCA demonstrated that aEEG that aEEG voltages were higher after CPB than before CPB, however the DHCA group had significantly lower aEEG voltages immediately after CPB. cTOI was unchanged in both groups immediately after CPB; however, after surgery, the DHCA group presented with higher cTOI and lower FTOE. The authors highlighted that neonates in the DHCA group were smaller than the non-DHCA group which may have influenced outcomes^96^. A single-center study evaluated a heterogeneous group of neonates with CHD with aEEG, NIRS and TCD and reported that rcSO_2_ and FTOE increased over the first 48 h postoperatively for all CHD groups, rcSO_2_ was lowest in SVP neonates at all time-points, and all neonates demonstrated impaired autoregulation during the postoperative period. However, there was no relationship between the rcSO_2_, FTOE and estimated autoregulation and new white matter injury or infarction on MRI^97^. A study of neonates who underwent CPB with and without deep hypothermic circulatory arrest (DHCA) for the repair of CHD evaluated the correlation of conventional EEG and aEEG noting that EEG was normal to mildly abnormal in 73% of neonates, moderately abnormal in 20% and markedly abnormal in 7%, while, using a single-channel aEEG, 3% were normal, 90% were moderately abnormal, and 7% were markedly abnormal. The authors concluded that a markedly abnormal aEEG was a good predictor of a moderately abnormal or markedly abnormal EEG^98^. The probability of a moderately or markedly abnormal EEG was 0.23 (0.17–0.30) with a moderate aEEG and the probability of a markedly abnormal EEG was 0.82 (0.53–0.95) with a markedly abnormal aEEG. A single-center study of 68 neonates with CHD reported that 50% had mild EEG background abnormalities in the preoperative period. Only one neonate had postoperative subclinical seizures, but most neonates in this cohort received benzodiazepine postoperatively. The lower seizure rate in this study may also explain why no clear associations between cerebral saturation monitored by NIRS and the presence of EEG seizures were found ^99^.

Few studies correlated multimodal monitoring with later outcome. A single-center study evaluated neonates with TGA and coarctation of the aorta highlighted that perioperative somatosensory evoked potentials (SSEPs) abnormalities were common in neonates with CHD, while brainstem auditory evoked potentials (BAEPs) were within normal limits in all neonates. Abnormal SSEPs and abnormal neurologic examinations correlated with abnormal neurologic findings assessed at 1 year of age with the Peabody Developmental Motor Scales and the Vineland Adaptive Behavior Scale^100^. A study of 20 consecutive term neonates with TGA without preoperative brain injury undergoing switch operation, demonstrated that rcSO_2_ measured by NIRS was <50% preoperatively in 80% of neonates with CPB and increased to normal values over the following 36 h except during intra-operative cardiac arrest when it decreased abruptly. After CPB, rcSO_2_ transiently decreased, followed by normalization of the values after 6–26 h later; recovery of the rcSO_2_ was longer for neonates with preoperative values <35%. There was no difference in aEEG recovery time between neonates with preoperative rcSO_2_ < 35% versus those with higher rcSO_2_. There were no seizures reported in this cohort and there was no statistically significant difference in neurodevelopmental outcome as measured by the BSID-2 at 30–36 months of age^101^. A case-control study in a heterogenous group of neonates with CHD and healthy age-matched controls ascertained features of sleep on a two-channel EEG as a marker of functional brain maturation and its correlation with rcSO_2_ measurements provided by NIRS. The authors reported that rcSO_2_ measured by NIRS were lower preoperatively in the d-TGA group versus other CHD groups. Neonates with TGA also spent less time in quiet sleep (high voltage slow wave) and more in quiet sleep (*tracé alternans*) as compared to controls; there was no change postoperatively. Sleep abnormalities were associated with a lower motor BSID-3 scores in neonates with CHD and specifically TGA, but not on cognitive or language scoring at 24 months^102^. These studies overall were very heterogeneous, lacked data synchronization, and did not demonstrate a consistent association of multimodal monitoring with neurodevelopmental outcome.

## Discussion

In this comprehensive scoping review, we have summarized published evidence on neuromonitoring of neonates with CHD. Most studies focused on the use of NIRS, followed by the use of EEG. There was geographic variability in the use of the different modalities, probably reflecting differences in resource availability and utilization. Most studies were single-center retrospective studies, which limits the generalizability of their findings. Most neuromonitoring studies also excluded premature neonates, low birthweight neonates, and neonates with chromosomal abnormalities, limiting generalizations to neonates without other comorbidities.

### NIRS

There were several studies describing the role of NIRS in neonates with CHD. Careful consideration should be given to the specific instrumentation used in each of the studies. Although many of the instruments used in the studies were commercially available, some were only available through research-based platforms. This will impact the applicability of the technology in different clinical settings. Although attractive in terms of cost, availability and portability, commercial NIRS devices lack precise quantitative capabilities. Measures provided by a commercial NIRS device are most useful as trend values of tissue oxygen saturation, where relative differences over time are considered. Interpretation of this output must consider oxygen consumption versus oxygen delivery. Additional limitations may include high susceptibility to ambient light contamination, variability based on sensor placement, heterogeneity of underlying tissue beds, skin pigmentation, and the need to account for common clinical variables that may affect NIRS values such as hemoglobin concentration, metabolic activity, temperature, and perfusion status. In contrast, other near infrared instruments such as frequency domain-NIRS or diffusion correlation spectroscopy are currently available in the research setting and compare absolute hemodynamic and oxygenation changes, providing discrete values such as cTOI. This distinction allows for comparisons within patients over time and between patients, and may have less susceptibility to ambient light, minimizing background noise signals. However, disadvantages include cost and lack of approval for clinical use compared to commercially available devices ^14^.

NIRS monitoring reasonably correlated with direct measurements of ScvO_2_ in neonates with CHD. There are transitory changes of rcSO_2_ in both SVP and TGA that are notable during intraoperative monitoring; however, these measurements were not clearly correlated with outcomes. Postoperative threshold values below 45% to 56% were associated with adverse hospital outcomes and adverse long-term neurodevelopmental outcomes. NIRS may be a marker of adverse short-term outcomes, although additional investigations remain necessary. At present, it remains unclear whether interventions in response to NIRS impacts outcome. The correlation of NIRS with long-term neurodevelopmental outcomes in neonates with CHD was only performed in single-center studies, with variable age at outcome assessment, variable near infrared instrumentation, and inclusion of heterogenous CHD phenotypes, which makes it challenging to generalize the results. Additionally, these studies analyzed a small number of subjects with a large number of clinical variables which may limit the conclusions drawn from these findings. Furthermore, other variables that may impact long term neurodevelopmental outcome such as maternal education and socioeconomic status were not systematically assessed in these studies. Only one study considered neonates with additional risk factors; the remainder of the studies excluded neonates with genetic syndromes, premature neonates, and low birthweight neonates with CHD to allow for long-term analysis of outcomes. Studies investigating NIRS did not examine its use in other co-morbidities frequently diagnosed in neonates with CHD such as strokes, new intracranial hemorrhages, or other intensive care complications; these would be additional important areas for future studies.

### EEG

EEG was useful in neonates with CHD to monitor seizures. In most studies, neonates were monitored for 48 h after surgery. Postoperative electrographic seizures were reported in 4–35% of neonates with CHD. Neonates were more likely to have electrographic seizures only, and, among those neonates with CHD and seizures, hypoxic ischemic encephalopathy (HIE), strokes or hemorrhages were the most common etiologies. This supports the routine postoperative use of continuous EEG in this population. Only one study examined the EEG background to identify changes preceding cardiac arrest; however, it remains unknown whether these changes could also be present in other neonates with CHD without cardiac arrest. The role of sedatives on EEG background was not explicitly explored in these studies. Most EEG studies included heterogeneous types of CHD; among the studies investigating only a single type of CHD, TGA was the most commonly studied. Questions regarding the association between EEG background and adverse neurodevelopmental outcomes have not yet been thoroughly explored in neonates with CHD, however a landmark study, the Boston Circulatory Arrest Study, has provided a signal for worsened neuropsychological long term outcomes in the setting of the presence of seizures^103,104^. Additionally, it is still unknown whether there are specific seizure burden thresholds associated with adverse neurodevelopmental outcomes in neonates with CHD and whether treating seizures will impact future neurodevelopmental outcome like in other critically ill-neonates.

### aEEG

In some geographical areas, the use of aEEG was more prevalent over the use of EEG to monitor seizures. Data were typically analyzed according to two widely used classification systems^79,105^. There was variability in the methodology of recording, since some studies used single-channel recordings versus two-channel recordings, which may limit generalizability of findings. Tracings were mostly reviewed by neonatologists rather than neurophysiologists. Most studies using aEEG analyzed CHD as a heterogeneous group. The studies were most often underpowered to demonstrate significant correlations with either neurodevelopmental outcome or neuroimaging. Only one prospective study was able to show an association between aEEG background and later IQ. Due to the variability in tools used to assess long-term neurodevelopmental outcomes, future research using a common assessment protocol would be valuable.

### TCD

Studies of TCDs were underpowered to draw conclusions. The heterogeneous nature of the neonates with CHD who were evaluated with this modality further limited the generalizability of the findings.

### Multimodal neuromonitoring techniques

There is emerging literature on the multimodal monitoring of neonates at risk for neurologic injury to better understand the underlying physiopathology of brain injury in CHD. Similarly to the studies that utilized NIRS, careful consideration should be given to the specific instrumentation used in each of the studies as the methodology will effect a significant impact on the result interpretation, and clinical availability. However, in the neonatal CHD population, the variability of the reported modalities, the complexity of the data, and the paucity of data integration made it challenging to truly incorporate and synchronize data into actionable models to fully explore the interrelationships of all the physiological variables.

### Knowledge gaps

Several research gaps were notable in this comprehensive evaluation of the literature on neuromonitoring of neonates with CHD: 1) At present, it remains unclear whether any one of these neuromonitoring techniques can reliably predict either adverse hospital outcomes or adverse neurodevelopmental outcomes; 2) Increased standardization of neurodevelopmental testing will be required to increase the generalizability of the data obtained from future studies; 3) Research is needed to best address geographic variation in resource availability and utilization for neonatal neurological monitoring; 4) Research on the integration and synchronization of multimodal monitoring in the evaluation of neonates with CHD will require complex data integration; 5) A framework for standardization of neuromonitoring techniques across centers through the use of clinical care guidelines should be evaluated; 6) Independent study of high-risk neonates in research studies is necessary to reflect the current care provided to all neonates with CHD, including premature neonates, low birth weight neonates or neonates with genetic disorders.

## Conclusions

In this comprehensive scoping review, we reviewed several neuromonitoring techniques available for use in neonates with CHD. NIRS may correlate with adverse hospital events and outcomes. EEG may identify subclinical seizures and status epilepticus and allow for the initiation of prompt and adequate treatment. aEEG may help support this practice when EEG is not available and may correlate with neurodevelopmental outcomes. TCDs may allow for more direct monitoring of cerebral blood flow. Multimodal monitoring may eventually allow for complex data integration increasing the precision of monitoring interpretations. However, multicenter studies with large sample size are needed to clarify reproducibility and validity of many of the single-center studies presented in this scoping review; and a framework for standardization of neuromonitoring techniques and standardized longitudinal follow-up will be required to define adequate interventions to this population.

## Supplementary Information

Supplementary Information 1

Supplementary Information 2

Supplemental Table 1

Supplemental Table 2

Supplementary table 3

## Data Availability

All data generated or analyzed during this study are included in this published article [and its supplementary information files].
