# Supplementary material for: Neuromonitoring practices for neonates with congenital heart disease: a scoping review
Source: Pediatr Res. 2024 Aug 25;97(5):1492–506. doi: 10.1038/s41390-024-03484-x (PMC12119335; doi:10.1038/s41390-024-03484-x)
Supplement: Supplementary file 3 — Supplemental Table 1 [file 41390_2024_3484_MOESM3_ESM.pdf]

Supplemental Table 1

Search: Search terms and strategies were developed closely with an experienced librarian (DN). The search strategy combined keywords and controlled vocabulary terms for the following concepts: infants, congenital heart diseases and neuromonitoring. We developed the search in Medline (OVID) and adapted it to the pre-specified databases.

A draft of our search strategy created in Medline (OVID) is provided below:

Ovid MEDLINE(R) and Epub Ahead of Print, In-Process, In-Data-Review & Other Non-Indexed Citations, Daily and Versions <1946 to March 03, 2023>		
1	exp Infant, Newborn/	667662
2	((Infant* and (preterm or term or premature or postmature)) or (Newborn* or Neonat*)).ti,ab.	486263
3	1 or 2	883859
4	exp Heart Defects, Congenital/	167822
5	exp Aortic Coarctation/	9790
6	exp Aortic Valve Stenosis/	50446
7	exp Hypoplastic Left Heart Syndrome/	2816
8	exp "Transposition of Great Vessels"/	8270
9	exp Truncus Arteriosus/	287
10	exp Univentricular Heart/	203
11	(((congenital adj2 (heart or cardiac)) and (disease* or defect* or abnormal* or malformation*)).ti,ab.	255844
12	("Single Ventricle Physiology" or "Hypoplastic left heart" or "transposition great arteries" or "truncus arteriosus" or "aortic valve stenosis" or "aortic coarctation" or "hypoplasia or interruption" or "left ventricular outflow tract" or "aortic arch obstruction" or "Univentricle heart").ti,ab.	18982
13	4 or 5 or 6 or 7 or 8 or 9 or 10 or 11 or 12	425433
14	exp Neurophysiological Monitoring/	2210
15	exp Diagnostic Techniques, Neurological/	435902
16	(Monitor* adj3 (brain or cerebral or multimodal or neonatal or neuro* or noninvasive or optical)).ti.	5546
17	("amplitude integrated electroencephalography" or "cerebral blood flow" or "cerebral hemoglobin oxygenation" or "cerebral near-infrared spectroscopy" or " cerebral near infrared spectroscopy" or "cerebral function monitor" or " cerebral oximetry electroencephalograph*" or EEG or "metabolic brain measurement*" or "Near-infrared spectroscopy" or "near infrared spectroscopy" or NIRS or "transcranial Doppler").ti,ab.	143003
18	14 or 15 or 16 or 17	505658
19	3 and 13 and 18	791
20	limit 19 to yr="1990 -Current"	702
