# Supplementary material for: Neuromonitoring practices for neonates with congenital heart disease: a scoping review
Source: Pediatr Res. 2024 Aug 25;97(5):1492–506. doi: 10.1038/s41390-024-03484-x (PMC12119335; doi:10.1038/s41390-024-03484-x)
Supplement: Supplementary file 4 — Supplemental Table 2 [file 41390_2024_3484_MOESM4_ESM.pdf]

## Supplemental table 2 Data characterization proforma

| A. Authorship characteristics | | |
|---|---|---|
| **Variable** | **Category** | **Explanation** |
| Author(s) | ☐ ____________________ | Please list. |
| Year of Publication | ☐ ____________________ | Please state. |
| Country | ☐ ____________________ | Please state the setting where the study took place. |

| B. General Study Characteristics | | |
|---|---|---|
| **Variable** | **Category** | **Explanation** |
| Study design | ☐ Case series<br>☐ Cohort<br>☐ Case Control<br>☐ Randomized controlled trial | Please select. |
| Objective(s) | ☐ ____________________<br>______________________ | Please briefly describe. |
| Population size | ☐ ____________________ | Please state. |
| Major sources of bias | | Please state |

| C. Description of Congenital Heart Disease | | |
|---|---|---|
| **Variable** | **Category** | **Explanation** |
| Type of Congenital Heart Disease | ☐ Single Ventricle Physiology<br>☐ Transposition of the great arteries<br>☐ Truncus arteriosus<br>☐ Coarctation of the aorta<br>☐ Left ventricular outflow obstruction<br>________________ | |

| D. Description of Neuromonitoring utilized | | |
|---|---|---|
| **Variable** | **Category** | **Explanation** |
| Format of technology | ☐ Electroencephalogram (EEG)<br>☐ amplitude integrated EEG (aEEG)<br>☐ Near Infrared Spectroscopy<br>☐ Transcranial Doppler<br>☐ Multimodal monitoring (>2 methods). | Please select all that apply. |
| Outcome measure(s) / assessment(s) / instrument(s) employed in study | ☐ ____________________ | Please briefly describe. |
| Duration of neuromonitoring study | ☐ ____________________ | Please state. |
| Indications for neuromonitoring study | ☐ | Please state. |

| E. Major findings | | |
|---|---|---|
| **Variable** | **Category** | **Explanation** |
| Major findings | ☐ ____________________<br>______________________<br>______________________ | Please describe. |
| Major challenges | ☐ ____________________<br>______________________ | Please describe. |
